# Metformin Treatment Shows Beneficial Effects on RTT-Associated Phenotypical Deficits in *Mecp2* T158M Male Mice

**DOI:** 10.3390/ph19040621

**Published:** 2026-04-15

**Authors:** Khatereh Saei Arezoumand, Ghanan Bin Akhtar, Ashraf Kadar Shahib, Jessica S. Jarmasz, Chris-Tiann Roberts, Abbas Rezaeian Mehrabadi, Carl O. Olson, Mojgan Rastegar

**Affiliations:** Department of Biochemistry and Medical Genetics, Max Rady College of Medicine, Rady Faculty of Health Sciences, University of Manitoba, Winnipeg, MB R3E 0J9, Canada

**Keywords:** Rett Syndrome, MeCP2, T158M mutation, metformin, anxiety, locomotion, neurodevelopmental disorders, phenotypical scoring, behavioral tests

## Abstract

**Background**: Rett Syndrome (RTT) is a progressive neurodevelopmental disorder caused by *MECP2* gene mutations. MeCP2 protein binding to methylated DNA is involved in normal brain development and function. T158M is a common RTT-associated mutation, where a threonine is replaced with a methionine, affecting protein function and stability. RTT has recently been identified as a neurometabolic disorder, with metformin emerging as a potential candidate drug. Metformin is a safe and accessible drug, commonly used for Type 2 diabetes. Our team previously studied the regulatory role of metformin on the expression of RTT-related genes/proteins using in vitro and in vivo approaches. However, the phenotypical and behavioral impact of metformin in transgenic mice carrying the common T158M mutation was not explored. **Methods**: Wild type (WT) and mutant *Mecp2^T158M^* (*Mecp2^tm4.1Bird^*) male mice were subjected to daily intraperitoneal injection of metformin for 20 days. The control mice received a daily intraperitoneal injection of the solvent. The main RTT-like phenotypical criteria were assessed daily. Behavioral tests included the open field test and elevated plus maze. **Results**: Behavioral tests indicated no significant effect of metformin on the anxiety levels, locomotion, and exploratory behaviors in the hemizygous male *Mecp2^T158M^* mice, despite our observation of increased anxiety levels in the WT counterparts. In hemizygous male *Mecp2^T158M^* mice, metformin treatment showed beneficial effects on RTT-like phenotypes, including breathing irregularities, gait abnormalities, hindlimb clasping, and overall total score. The positive effect of metformin was also observed on the body weight in the hemizygous male *Mecp2^T158M^* mice. **Conclusions**: Our findings provide evidence for potential therapeutic effects of metformin for MeCP2-associated neurological disorders.

## 1. Introduction

Rett Syndrome (RTT) is a rare and X-linked neurodevelopmental disorder. After an apparently normal period of development, typically within 6–18 months after birth, the symptoms emerge, including developmental regression, disappearance of learned skills, such as speech, purposeful use of hands, motor coordination, as well as intellectual disabilities, accompanied by gait abnormalities and seizures [1,2,3,4]. Over 95% of RTT cases are caused by de novo mutations in the *MECP2* gene, which encodes the Methyl-CpG-Binding Protein 2 (MeCP2) [2]. MeCP2 is an epigenetic reader of methylated DNA, playing an essential role in regulating gene expression and maintaining neuronal structure and function. While MeCP2 was primarily considered a transcriptional repressor, it is now recognized for its dual functionality as an activator and an inhibitor of gene expression, in a context-dependent manner. Additionally, MeCP2 is involved in maintaining chromatin architecture and condensation, RNA splicing, mitochondrial function, and synaptic plasticity, all of which are crucial for proper neuronal maturation and function [3,5,6,7,8,9]. The T158M missense mutation is one of the most prevalent MeCP2 mutations detected in RTT patients. This loss-of-function mutation affects the DNA-binding domain of MeCP2 protein and is associated with reduced protein stability and/or expression in specific brain regions. Consequently, this mutation relates to important molecular, phenotypical, and behavioral deficits, including altered expression of synaptic proteins and/or synaptic activity [10,11,12]. Hemizygous male *Mecp2^T158M^* mice display a more severe phenotype compared to their female *Mecp2^T158M^* littermates [11,12], implying that the associated symptoms are influenced by sex, a fact that is not surprising, as the *MECP2/Mecp2* gene is located on the X chromosome [2]. Such evidence underlines the complexity of RTT pathophysiology, highlighting the need for research studies that consider sex as a biological factor. Despite substantial progress in understanding the molecular mechanisms underlying RTT, no definite cure is currently available, and existing therapies primarily address RTT symptoms.

Despite the absence of extensive, effective and accessible RTT therapies, recent studies have suggested that the anti-diabetic drug metformin may offer neuroprotective benefits for RTT [13,14]. Metformin is suggested to act through signaling molecules such as AMP-activated protein kinase (AMPK) that controls energy balance. AMPK activation and its interaction with the mammalian Target of Rapamycin (mTOR) pathway also regulate neuronal energy homeostasis, protein synthesis, and cellular growth, which are disrupted in Rett Syndrome [15,16,17,18]. However, further studies investigating the potential therapeutic effect of metformin in the context of RTT are warranted.

Our preceding studies show that metformin treatment may have some potential therapeutic effect for MeCP2-associated disorders [14,19,20]. Here, we aimed to investigate the potential therapeutic effects of metformin treatment in hemizygous male *Mecp2^T158M^* mice. We hypothesized that metformin may moderately and partially alleviate RTT-associated phenotypical and behavioral deficits that are affected in *Mecp2^T158M^* mice. Our data may provide some insight into a potential therapeutic approach for RTT and other MeCP2-associated disorders.

## 2. Results

### 2.1. Metformin Did Not Alleviate Brain Size Deficits in the Hemizygous Male Mecp2^T158M^ Mice

**Figure 1:**
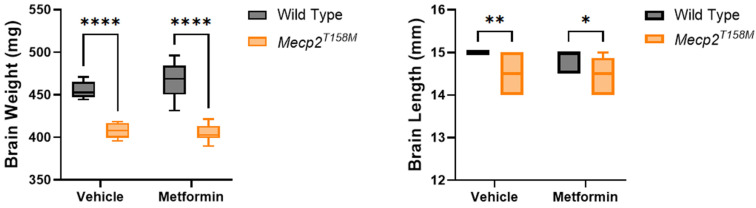
Brain weight (mg) and length (mm) of male wild type (WT) and hemizygous *Mecp2^T158M^* mice (8–9 weeks old) treated with metformin and compared to vehicle-treated control mice. Brain weight and length were significantly lower in vehicle-treated hemizygous male *Mecp2^T158M^* mice compared to their WT controls, reflecting RTT-like neuroanatomical deficits. Data is presented as box plots showing the mean± SEM as well as the minimum and maximum values. For all groups, a minimum of five mice is used in these experiments. The statistical analysis was completed using a Two-way ANOVA. Biological replicates include male WT vehicle-treated mice *N* = 5, male WT metformin-treated mice *N* = 8, hemizygous male *Mecp2^T158M^* vehicle-treated mice *N* = 9; hemizygous male *Mecp2^T158M^* metformin-treated *N* = 11 (brain weight) and *N* = 12 (brain length). Significance is reported by * *p* < 0.05, ** *p* < 0.01, and **** *p* < 0.0001.

Neurodevelopmental disorders such as RTT are commonly linked with structural brain anomalies, including decreased overall brain size and weight, as well as reduced volume in particular brain regions like the striatum, thalamus, cortex, and white matter, detected in both patients as well as RTT mouse models [12,21,22]. To investigate whether MeCP2 mutation induces similar changes and to assess the effect of metformin treatment (250 mg/kg body weight, daily intraperitoneal (IP) injection for 20 days), we measured the brain weight and length in WT and hemizygous male *Mecp2^T158M^* mice at the end of the study, when mice are most symptomatic (between 8 and 9 weeks of age). As presented in Figure 1, the vehicle-treated (solvent: Phosphate-Buffered Saline (PBS)) hemizygous male *Mecp2^T158M^* mice showed a significant decrease in brain weight and length in comparison to WT controls. The average brain weight of vehicle-treated hemizygous male *Mecp2^T158M^* mice was lowered by ~10.6% compared to vehicle-treated male WT mice, confirming a genotype-dependent reduction in the brain mass. Similarly, brain length was significantly lower in the vehicle-treated hemizygous male *Mecp2^T158M^* mice compared to the vehicle-treated male WT mice. Among the experimental mutant mice, these deficits were not restored following metformin treatment.

### 2.2. Metformin May Have a Positive Effect on the Anxiety-like Behavior in the Hemizygous Male Mecp2^T158M^ Mice

**Figure 2:**
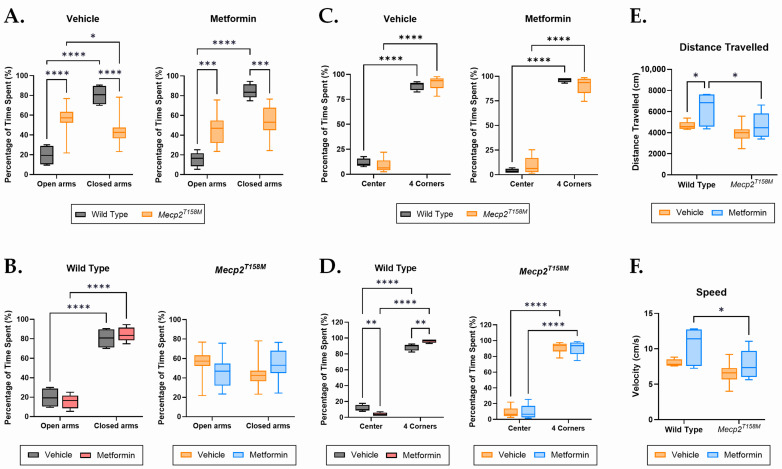
Behavior testing results comparing the wild type and hemizygous male *Mecp2^T158M^* mice (average of 8–9 weeks). Anxiety-like behaviors were measured by the percentage of time spent in the open arms and on the closed arms over the course of 10 min in the Elevated Plus Maze (EPM). (**A**,**B**) The percentage of time spent by the vehicle- and metformin-treated groups. Locomotion and anxiety-like behaviors were assessed simultaneously in the Open Field Test (OFT). We placed the mice in the field facing one of the walls and let them explore for 10 min (600 s). (**C**,**D**) The percentage of time spent in the center versus all four corners of the arena is shown. (**E**) Total distance moved and (**F**) average velocity in the OFT are also shown. Minimum–maximum box plots with the mean ± SEM are shown for each group. For all groups, a minimum of 5 mice is used in these experiments. For EPM, male WT vehicle-treated mice *N* = 6, male WT metformin-treated mice *N* = 6, hemizygous male *Mecp2^T158M^* vehicle-treated mice, *N* = 10, hemizygous male *Mecp2^T158M^* metformin-treated mice *N* = 9. For OFT, male WT vehicle-treated mice *N* = 5, male WT metformin-treated mice *N* = 5 (percentage of time)/*N* = 6 (distance and velocity), hemizygous male *Mecp2^T158M^* vehicle-treated mice *N* = 10, hemizygous male *Mecp2^T158M^* metformin-treated mice *N* = 8 (percentage of time)/*N* = 9 (distance and velocity). The reported data are analyzed by Two-way ANOVA, and with significance of * *p* < 0.05, ** *p* < 0.01, *** *p* < 0.001, and **** *p* < 0.0001.

Change in anxiety level is an obvious characteristic of the neurological impairments detected in both RTT patients and RTT mouse models [23]. To evaluate the effects of metformin and vehicle treatment on anxiety, we performed the Elevated Plus Maze (EPM) test on male WT and hemizygous *Mecp2^T158M^* mice. This test was used to evaluate the possible therapeutic effects of metformin on RTT-associated behavioral deficits, specifically their anxiety-like behavior. We previously showed that hemizygous male *Mecp2^T158M^* mice display reduced anxiety-like behavior, spending a significantly higher amount of time in the open arms and a lower amount of time in the closed arms, when compared with their WT age-matched control mice [11]. In the present study, vehicle-treated hemizygous male *Mecp2^T158M^* mice continued to display a similar behavior, spending significantly more time in the open arms than in the closed arms (**** *p* < 0.0001), while WT control mice spent significantly more time in the closed arms relative to open arms (**** *p* < 0.0001) baseline of normal anxiety levels in males at this age (Figure 2A,B). After 20 days of metformin treatment, the time spent in the open versus the closed arms remained the same for WT, with no significant change in the hemizygous male *Mecp2^T158M^* mice (Figure 2B).

### 2.3. Differential Responses to Metformin in the Wild Type and Hemizygous Male Mecp2^T158M^ Mice During the Open Field Test

Both WT and hemizygous male *Mecp2^T158M^* mice in our experimental groups spent significantly more time in the four corners of the open field than in the center (**** *p* < 0.0001; Figure 2C), indicating reduced exploratory behavior and increased thigmotaxis. When comparing the effect of metformin treatment among WT mice alone, it appears metformin significantly reduced the time spent in the center and increased the time spent in the four corners when compared to the vehicle (** *p* < 0.01; Figure 2D), while no change was observed among hemizygous male *Mecp2^T158M^* mice (Figure 2D). Although the EPM is the main measure of anxiety in this study, this finding indicates that metformin may increase anxiety-like behavior in male WT mice, particularly in the Open Field Test, as one can interpret the time spent in the defined areas of the OFT as a measure of anxiety.

In this study, the OFT was primarily used to evaluate locomotion, measured by the total distance traveled as well as the speed. In vehicle-treated mice, both WT and hemizygous male *Mecp2^T158M^* mice traveled a similar distance (Figure 2E) and at a similar speed (Figure 2F). Interestingly, WT males showed a significant increase in the distance travelled (* *p* < 0.05; Figure 2E) when treated with metformin. This suggests increased locomotion and further supports higher anxiety among WT males treated with metformin. However, the hemizygous male *Mecp2^T158M^* mice did not show any significant change with metformin treatment (Figure 2E). These results further highlight a change in anxiety levels as well as an increase in locomotion, among WT males.

### 2.4. Metformin Treatment Had a Significant Positive Effect on the RTT-like Phenotypes in the Hemizygous Male Mecp2^T158M^ Mice

The potential therapeutic effects of metformin on RTT-like symptoms in hemizygous male *Mecp2^T158M^* mice and their WT littermates were evaluated using a standard phenotypical scoring system over 20 days. Here, we measured six phenotypical parameters, including activity/mobility, gait, hindlimb clasping, tremor, general condition/appearance, and breathing irregularities. Each parameter was evaluated daily on a 0–2 severity scale (0 = absence of the impaired phenotype—normal phenotype observed in WT mice, 1 = moderate level of impaired phenotype, and 2 = severe level of impaired phenotype), based on criteria previously established in RTT mice [24], and previously used by us [11].

**Figure 3:**
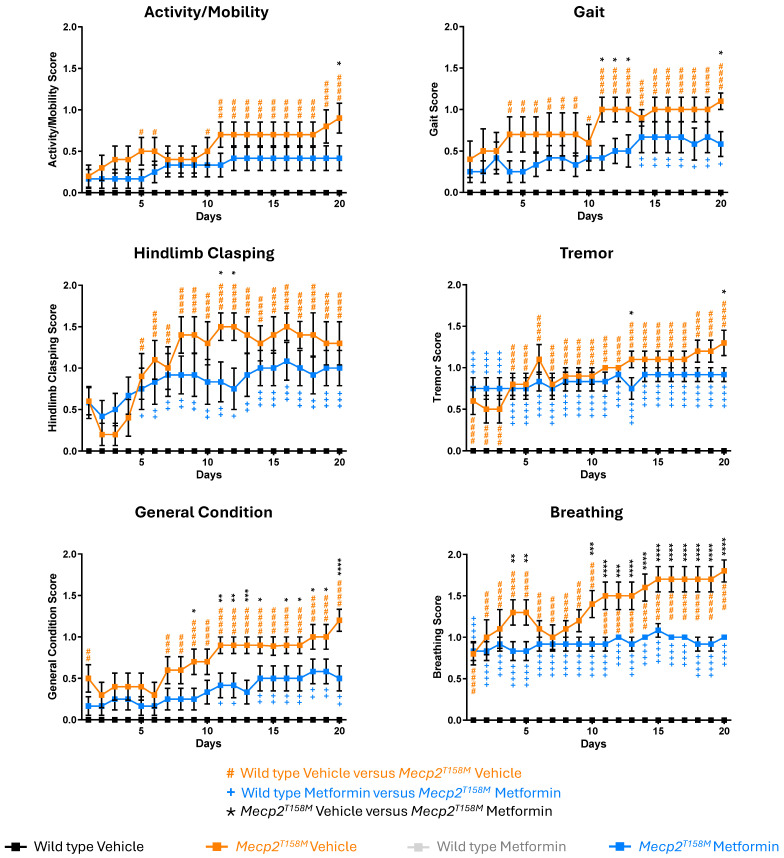
Daily phenotypical scoring of RTT-like symptoms in the hemizygous male *Mecp2^T158M^* and wild type male mice treated with vehicle or metformin. Daily scores for six indicated monitored phenotypical criteria over a 20-day treatment period, where wild type (WT) mice have a daily score of zero (vehicle- and metformin-treated) and hemizygous male *Mecp2^T158M^* mice have scores ranging from 0 to 2 (vehicle- and metformin-treated). Data are shown as mean ± SEM. Male WT vehicle-treated mice *N* = 10, WT metformin-treated mice *N* = 8, hemizygous male *Mecp2^T158M^* vehicle-treated mice, *N* = 10, hemizygous male *Mecp2^T158M^* metformin-treated mice *N* = 12. The reported data are analyzed by Two-way ANOVA, showing WT versus *Mecp2^T158M^* vehicle (orange hashtag symbol), male WT metformin-treated mice versus hemizygous male *Mecp2^T158M^* metformin-treated mice (blue plus symbol) and hemizygous male *Mecp2^T158M^* vehicle-treated mice versus metformin-treated mice (black star symbol). Significance of the results is reported with # *p* < 0.05, ## *p* < 0.01, ### *p* < 0.001, #### *p* < 0.0001, + *p* < 0.05, ++ *p* < 0.01, +++ *p* < 0.001, ++++ *p* < 0.0001, * *p* < 0.05, ** *p* < 0.01, *** *p* < 0.001, and **** *p* < 0.0001.

**Figure 4:**
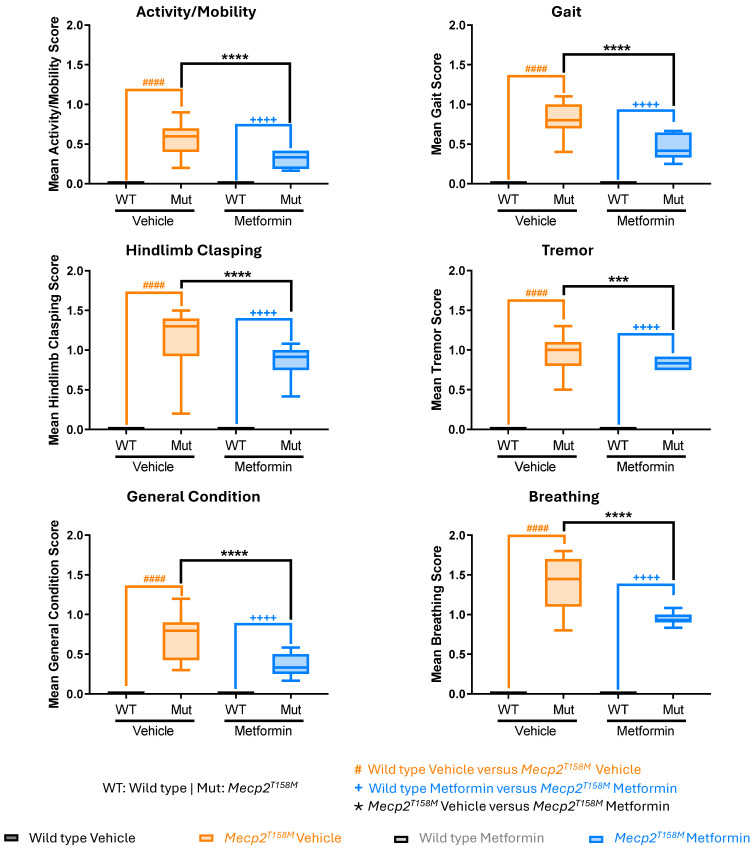
The overall 20-day mean of RTT-like symptoms in the hemizygous male *Mecp2^T158M^* and wild type mice treated with vehicle or metformin. Mean scores for six indicated monitored phenotypical criteria over a 20-day treatment period, where wild type (WT) male mice have a daily score of zero (vehicle- and metformin-treated) and hemizygous male *Mecp2^T158M^* mice have scores ranging from 0 to 2 (vehicle- and metformin-treated). Data are shown as mean ± SEM. Male WT vehicle-treated mice *N* = 10, male WT metformin-treated mice *N* = 8, hemizygous male *Mecp2^T158M^* vehicle-treated mice, *N* = 10, hemizygous male *Mecp2^T158M^* metformin-treated mice *N* = 12. The reported data are analyzed by Two-way ANOVA, showing male WT versus hemizygous male *Mecp2^T158M^* vehicle-treated mice (orange hashtag symbol), male WT versus hemizygous male *Mecp2^T158M^* metformin-treated mice (blue plus symbol) and hemizygous male *Mecp2^T158M^* vehicle-treated versus metformin-treated mice (black star symbol). Significance of the results is reported with #### *p* < 0.0001, ++++ *p* < 0.0001, *** *p* < 0.001, and **** *p* < 0.0001.

As expected, the daily scores for vehicle-treated hemizygous male *Mecp2^T158M^* mice were higher in all phenotypical parameters compared to WT controls (Figure 3 and Figure 4), demonstrating a worsening condition in the mutant mice. Cumulative scores for every single criterion over the 20 days were significantly higher in the vehicle-treated hemizygous male *Mecp2^T158M^* mice (#### *p* < 0.0001), representing a significant decline in motor function, respiratory health, and overall condition (Figure 4). Remarkably, gait abnormalities, hindlimb clasping, and tremor appeared as key symptoms, reflecting the typical beginning and increase in motor deficits characteristic of RTT pathology [25,26]. Likewise, breathing abnormalities, a deterioration in general condition, and decreased activity levels also worsened over time (Figure 4).

**Figure 5:**
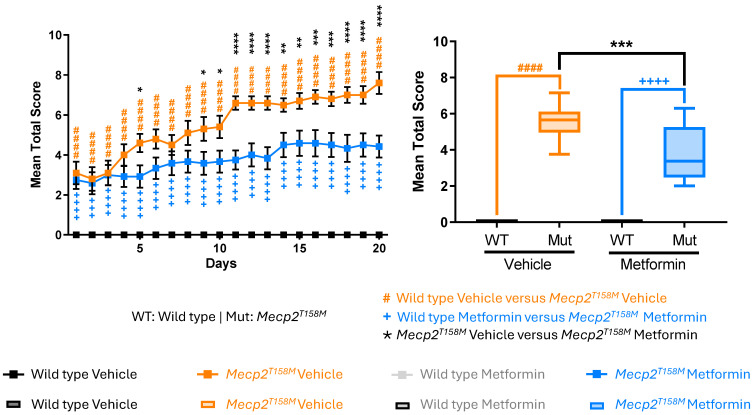
Cumulative (total) phenotypical scoring of RTT-like symptoms in the hemizygous male *Mecp2^T158M^* and wild type mice treated with vehicle or metformin. Wild type (WT) mice have a daily score of zero (vehicle- and metformin-treated) and *Mecp2^T158M^* scores range from 0 to 2 (vehicle- and metformin-treated). Cumulative (total) 20-day scores for each parameter and the overall composite severity score, presented as bar graphs and day-wise graphs. Statistical analysis was done by Two-way ANOVA, showing male WT versus hemizygous male *Mecp2^T158M^* vehicle-treated mice (orange hashtag symbol), male WT versus hemizygous male *Mecp2^T158M^* metformin-treated mice (blue plus symbol) and hemizygous male *Mecp2^T158M^* vehicle-treated mice versus metformin-treated mice (black star symbol). Data are shown as mean ± SEM. WT vehicle *N* = 10, WT metformin *N* = 8, *Mecp2^T158M^* vehicle, *N* = 10, *Mecp2^T158M^* metformin *N* = 12. Significance is indicated with #### *p* < 0.0001, +++ *p* < 0.001, ++++ *p* < 0.0001, * *p* < 0.05, ** *p* < 0.01, *** *p* < 0.001, and **** *p* < 0.0001.

Importantly, metformin-treated hemizygous male *Mecp2^T158M^* mice showed significantly lower scores across all assessed phenotypical criteria when compared to vehicle-treated mice (Figure 3 and Figure 4), suggesting a positive effect on their phenotypical criteria. Prominent positive effects were also detected in respiratory function, with breathing irregularities showing a substantial impact from approximately day 10 forward, and these positive effects were maintained throughout the treatment. Moreover, positive effects were detected across all six criteria that were measured, with statistical significance ranging from * *p* < 0.05 to **** *p* < 0.0001 in daily scoring analysis (Figure 3). Notably, consideration of all 20 days of phenotypical monitoring together indicated significantly lower scores of all six parameters over the 20 days in metformin-treated hemizygous male *Mecp2^T158M^* mice compared to vehicle-treated controls with the same genotype (*** *p* < 0.001 to **** *p* < 0.0001) (Figure 4). Analysis of daily phenotypical scoring showed a clear beneficial impact of metformin on symptom severity (indicated by lower phenotypical scoring values). These results align with the neuroprotective, anti-inflammatory, and metabolic regulatory effects of metformin, which have been recognized in other models of neurodegenerative and neurodevelopmental disorders [18,27]. The total score (mean cumulative score of all six evaluated phenotypical criteria) was also calculated and proved to be significantly lower (*** *p* < 0.001) in the metformin-treated hemizygous male *Mecp2^T158M^* mice compared to the vehicle-treated control mice with the same genotype (Figure 5).

**Figure 6:**
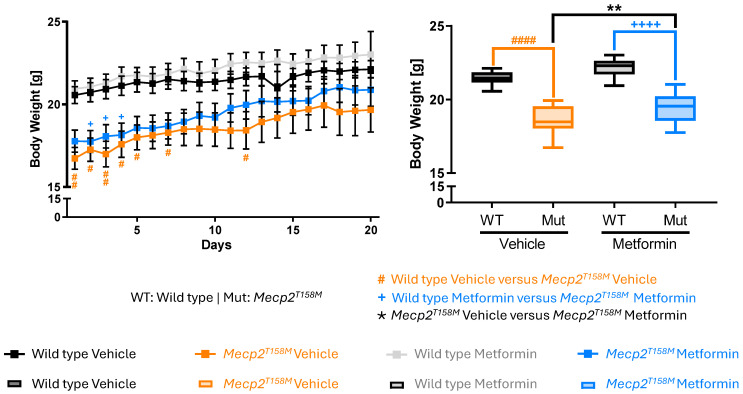
Daily body weight measurements in hemizygous male *Mecp2^T158M^* and wild type mice treated with vehicle or metformin. Daily body weight (in grams) was measured over the 20-day treatment period. Results are represented as mean ± SEM. Male wild type (WT) vehicle-treated mice *N* = 10, male WT metformin-treated mice *N* = 8, hemizygous male *Mecp2^T158M^* vehicle-treated mice, *N* = 10, hemizygous male *Mecp2^T158M^* metformin-treated mice *N* = 12. Statistical analysis was done by Two-way ANOVA, showing male WT versus hemizygous male *Mecp2^T158M^* vehicle-treated mice (orange hashtag symbol), male WT versus hemizygous male *Mecp2^T158M^* metformin-treated mice (blue plus symbol) and hemizygous male *Mecp2^T158M^* vehicle-treated mice versus metformin-treated mice (black star symbol). Significance of the results is indicated with # *p* < 0.05, ## *p* < 0.01, #### *p* < 0.0001, + *p* < 0.05, ++++ *p* < 0.0001, and ** *p* < 0.01.

Daily monitoring of body weight was also performed as a means of evaluating the overall health status of mice in different experimental groups. Both male WT and hemizygous *Mecp2^T158M^* mice demonstrated an increase in their body weight over the course of 20 days. The mean bodyweight of these mice showed that both the vehicle- and metformin-treated hemizygous male *Mecp2^T158M^* mice have significantly lower body weight compared with their control WT male mice (#### *p* < 0.0001, ++++ *p* < 0.0001). However, we also observed that metformin significantly increased the weight in the hemizygous male *Mecp2^T158M^* mice compared to their vehicle-treated counterparts (** *p* < 0.01) (Figure 6).

## 3. Discussion

MeCP2 plays an important role in brain development, as well as neuronal differentiation, maturation, and function throughout life. The MeCP2 mutation at amino acid 158 within the methyl-binding domain severely impairs the proper function of this protein domain, as well as protein instability in the brain [12,28,29]. This mutation is among the most prevalent mutations in RTT and is strongly linked with multiple pathophysiological results [29,30], emphasizing potential paths for targeted therapeutic interventions.

Our results for hemizygous male *Mecp2^T158M^* mice further represent these phenotypical outcomes, showing regular reductions in brain weight and size across symptomatic stages. Indeed, these changes closely reflect those observed in human RTT patients, where total brain size is reduced without apparent neuronal loss [31,32]. It is possible that defects in neuronal growth or dendritic arborization, instead of neuronal cell death, might cause the structural brain impairments in RTT. The essential role of MeCP2 in maintaining proper neuronal synaptogenesis emphasizes its significance in brain development, neuronal function, and involves MeCP2 homeostasis regulation, which is affected in Rett Syndrome [3,33]. Our in vivo and in vitro studies previously suggested that metformin may induce *Mecp2* promoter activity, *MECP2* transcripts and/or MeCP2 protein levels in the brain tissues/and or in brain cells [14,19,20]. In this study, metformin treatment did not rescue the reductions in brain weight or length observed in the MeCP2 T158M mice. This dissociation between structural outcomes and phenotypical scoring with positive effects may suggest that metformin does not reverse global neurodevelopmental deficits in this mouse model, at least in male mice at 8–9 weeks of age. Instead, the effects of metformin on phenotypical scores may reflect functional modulation of neuronal circuits rather than large-scale anatomical restoration. Gross brain mass measurements may not be sufficiently sensitive to detect cellular or synaptic-level changes that influence motor control, respiratory regulation, or anxiety-like responses. It is therefore possible that metformin exerts its effects through mechanisms that improve neuronal function or network stability without correcting overall brain growth. However, further investigation into specific molecular mechanisms may be the focus of future research.

We observed that the vehicle-treated hemizygous male *Mecp2^T158M^* mice displayed RTT-like symptoms, including respiratory irregularities, motor deficits, and reduced anxiety-like behaviors compared to WT mice. Phenotypically, metformin treatment positively affected the symptoms, while behaviorally, no significant impact was observed. These positive effects were significant in activity/mobility, gait, tremor, hindlimb clasping, breathing, and general condition, suggesting the beneficial effects of the involved molecular pathways. In addition, our detected increase in the anxiety levels and locomotion among WT mice treated with metformin may suggest possible changes in neural circuitry, associated with increased risk-aversion [34].

Our findings may also support the therapeutic potential of metformin as a metabolic modulator for RTT. Our previous studies indicated that MeCP2 levels can be increased in a brain region-dependent manner in male mice [14]. It is possible to speculate that a potential increase in MeCP2 T158M may have been reached by metformin treatment to help the phenotypical criteria of hemizygous male *Mecp2^T158M^* mice, observed here. Improved phenotype in a MeCP2 T158M mouse model, through genetic approaches and due to the increased protein level of the mutated protein, has been well documented [12]. Our results may suggest that investigating a combination of drug therapy strategies to target downstream metabolic and/or molecular pathways might potentially address the molecular, phenotypical, and behavioral deficits of Rett Syndrome.

Although our study clearly suggests a beneficial effect in phenotypical impairment of hemizygous male *Mecp2^T158M^* mice, certain limitations may exist. One aspect to complement our research would be a comprehensive molecular and cellular assessment of different experimental groups to address the effect of metformin in different parts of the brain. As the primary target of metformin is the liver, analyzing the metabolic aspects of liver function would also be of interest. Based on previous research that has been done on the role of metformin in the brain, several plausible molecular mechanisms could be speculated. Metformin is a well-established activator of AMPK, a regulator of cellular metabolism [35]. Apart from its role in mitochondrial function, AMPK is suggested to be involved in synaptic plasticity and neuronal survival [18]. AMPK functions downstream of the mTOR pathway, which controls fundamental cellular processes that are interrupted in the context of RTT, such as cellular growth, protein synthesis and synaptic activity [36,37,38,39]. Furthermore, dysregulation of mTOR and related signaling pathways has been reported in RTT and other neurodevelopmental disorders [37,38,40], and metformin has been shown to normalize these pathways in models such as Fragile X Syndrome, where it restores both molecular markers and behavioral deficits [41].

In addition to AMPK/mTOR signaling, emerging evidence suggests that metformin may exert beneficial effects through the modulation of mitochondrial function and oxidative stress, both of which are altered in RTT. Previous studies in RTT mouse models have demonstrated that metformin can restore mitochondrial biogenesis and function, as indicated by increased ATP production and normalization of key regulators [13,42]. Beyond these pathways, metformin has also been reported to enhance neurogenesis, promote synaptic plasticity, and reduce neuroinflammation by suppressing pro-inflammatory signaling pathways, thereby limiting microglial and astrocyte activation [27]. Furthermore, our previous work has demonstrated that metformin may increase *MECP2* transcript levels in an isoform-specific manner and modulate downstream targets such as Brain-derived neurotrophic factor (*BDNF*) [20]. The produced BDNF protein plays a critical role in neuronal development and synaptic plasticity [43]. Additionally, metformin induces *Mecp2* promoter activity [19] that may lead to increased MeCP2 protein levels in the brain. Increased levels of MeCP2 and/or BDNF in the brain are suggested to have potential therapeutic effects for RTT [12,44]. Collectively, these studies suggest that metformin may exert multifaceted neuroprotective effects through metabolic, transcriptional, and anti-inflammatory mechanisms. However, the accuracy of these theories remains to be further explored.

Future research may also focus on evaluating treatment efficacy in heterozygous female models and investigating targeted therapies, such as cell type-specific and brain region-specific interventions. While metformin alone may not provide a cure for RTT, its role as a metabolic modulator deserves continued exploration, specifically in combination with other therapies aimed at restoring the neuronal balance for Rett Syndrome. Future studies to include the heterozygous female *Mecp2^T158M^* mice would be beneficial to study the effects of metformin across both sexes. Moreover, investigating treatment intervals and other RTT mouse models may offer additional insights into the therapeutic effects of metformin for Rett Syndrome.

## 4. Materials and Methods

### 4.1. T158M Mice and Treatments

All of our reported experimental tests on live mice were reviewed and approved by the University of Manitoba Animal Research Ethics Board (protocol numbers 18-053 and 23-019). Mice were housed in a facility with controlled temperature as well as free access to food and water. We purchased the T158M transgenic mice (B6.129P2(Cg)-*Mecp2^tm4.1Bird/J^;* strain 026762) from Jackson’s Laboratory. The University of Manitoba Genetic Modeling Center (GMC) maintained the T158M colony on a C57BL/6 background. Mutant (*Mecp2^T158M^*) animals were identified by genotyping with specific primer sets, as previously described [11].

A total of 6 animals treated with metformin (1 male WT, 5 hemizygous male *Mecp2^T158M^* mice) were eliminated from the analysis due to reaching the humane endpoint (≥15% total body weight loss, as defined in our approved animal protocol) or due to fight wounds. In one case, a hemizygous male *Mecp2^T158M^* mouse treated with metformin was found dead. The total number of animals in experimental groups varied, with a minimum of 5 mice in each group. A detailed number of analyzed mice for each experiment is included in the figure legends. Male wild type and hemizygous male *Mecp2^T158M^* mice were randomly placed into vehicle-treated and metformin-treated experimental groups. Mice were weighed and subjected to daily intraperitoneal injection of the vehicle/solvent (PBS) or metformin (EMD Millipore Crop, St. Louis, MO, USA, 317240) at a dosage of 250 mg/kg body weight for 20 days. We chose a dose of 250 mg/kg in mice, as it corresponds to the daily recommended dose of 20 mg/kg in humans [45,46,47]. Metformin was reconstituted and diluted in PBS. Both metformin and PBS were passed through a 0.22 µm filter before being loaded into syringes to ensure sterility. This study was not conducted blindly, as the mice would be scored for phenotype and then subsequently injected with PBS or metformin right after being weighed to determine the correct volume to administer (based on body weight). Wild type male mice were the baseline comparison and always received a score of zero, which reflects normal phenotypical criteria [24]. The phenotyping scoring sheets included the genotype and treatment group of the mouse that was randomly placed in the experimental groups, and data sheets were used for data collection consistency.

This study began with male mice at the age of 5–6 weeks and ended by 8–9 weeks. For 20 days, all the animals were monitored daily and scored for RTT phenotype, as previously described [11]. This included activity, gait, tremor, hindlimb clasping, breathing, and general condition/appearance. Briefly, activity and gait were assessed by how quickly the mouse walked when placed on a tabletop and how wide their stance was, as well as how low their rump/pelvis was to the table. Hindlimb clasping (positioning and movement of hind-feet) was assessed while the mouse was suspended in the air by the base of their tail. Tremor (waves of what appeared as shivering), and breathing (rapid versus slowed or labored) were observed while the mouse sat in the palm of your hand. General condition (appearance) was assessed by simply looking at the mouse and observing changes in the shine of their coat, level of grooming, and clearness of the eyes. A score of 0, 1, or 2 was given accordingly. Phenotypical scoring of the animals throughout the study was completed daily. After 20 days, mice were euthanized (by CO_2_ overdose), as previously described [11]. The whole brain was removed and subsequently weighed (in milligrams) and the length measured (in millimeters).

### 4.2. Behavioral Testing

Behavioral testing was conducted during the last three days of the treatment and was done in a partially blinded manner (genotype was known, but not the treatments). Mice were placed in a darkened tent with infrared lighting and tracked using EthoVision XT software (v16.0.1538, Noldus) for precise movement tracking. The testing apparatuses were cleaned with 70% ethanol between subjects, and mice had a 24 h rest period between tests to minimize stress [11]. EPM was applied to study anxiety-like behavior in mice by calculating the time that they spent in the open versus closed arms of the equipment [48]. Mice were placed at the center of the maze and recorded for 10 min. The key metrics were the amount of time spent in each arm (open versus closed) and the total distance traveled. The percentage of time spent in open and closed arms was calculated as an indicator of anxiety. For the OFT study, mice were positioned in a 60 cm × 60 cm arena and observed for 10 min to measure locomotion and anxiety. Key metrics included total distance traveled, velocity, and time spent in the center versus the corners of the arena. The percentage of time spent in each area was determined.

### 4.3. Statistics for Different Experiments

All data were graphed using GraphPad Prism Software (version 10). Brain weight and length data were analyzed by Two-way ANOVA with Tukey’s post hoc test. Behavior data were analyzed by Two-way ANOVA with an uncorrected Fisher’s LSD post hoc test after outlier identification. The values that were more than ±1.5 standard deviations above and below the mean were considered outliers [49] for brain criteria and behavior data. No outliers were identified or removed from the phenotypical scoring analysis (Figure 3, Figure 4, Figure 5 and Figure 6). No more than 2 outliers were identified and removed for each group (Figure 1 and Figure 2). Body weight and phenotypical scoring data were analyzed by Two-way ANOVA, with a Sidak’s post hoc test for the daily graphs and a Tukey’s post hoc test for the cumulative graphs.

## 5. Conclusions

In this current study, we evaluated the potential benefits of metformin treatment on moderating RTT-like phenotypes, as well as evaluating behavior and brain characteristics in hemizygous male *Mecp2^T158M^* mice compared to WT counterparts. A significant reduction in brain mass was observed in the vehicle-treated mutant male mice compared to WT male control mice, which was not positively affected by metformin. Moreover, hemizygous male *Mecp2^T158M^* mice showed a range of RTT-like phenotypical and behavioral symptoms. The beneficial effects that were observed on the phenotypical criteria in the hemizygous male *Mecp2^T158M^* mice with metformin treatment may suggest a partial restoration of neuronal function. Our results support earlier preclinical research on potential repurposing of metformin for neurodevelopmental disorders such as Rett Syndrome.

## Data Availability

The original contributions presented in this study are included in the article. Further inquiries can be directed to the corresponding author.

## Abbreviations

The following abbreviations are used in this manuscript:

AMPK	AMP-activated protein kinase
*BDNF*	Brain-derived neurotrophic factor (gene)
BDNF	Brain-derived neurotrophic factor (protein)
CCAC	Canadian Council on Animal Care
CHRIM	Children’s Hospital Research Institute of Manitoba
EPM	Elevated Plus Maze
GMC	Genetic Modeling Center
IP	Intraperitoneal
MeCP2	Methyl-CpG-Binding Protein 2 (protein)
*MECP2*	human gene
*Mecp2*	mouse gene
mTOR	mammalian Target of Rapamycin
OFT	Open Field Test
PBS	Phosphate-Buffered Saline
RTT	Rett Syndrome
SEM	Standard Error of the Mean
WT	Wild type
